# Transition from spiral wave chimeras to phase cluster states

**DOI:** 10.1038/s41598-020-64081-6

**Published:** 2020-05-08

**Authors:** Jan Frederik Totz, Mark R. Tinsley, Harald Engel, Kenneth Showalter

**Affiliations:** 1 Department of Mechanical Engineering, Massachusetts Institute of Technology, Cambridge, MA 02139 USA; 2 Department of Mathematics, Massachusetts Institute of Technology, Cambridge, MA 02142 USA; 3 C. Eugene Bennett Department of Chemistry, West Virginia University, Morgantown, WV 26506-6045 USA; 4 Institut für Theoretische Physik EW 7-1, TU Berlin, Hardenbergstr. 36, 10623 Berlin, Germany

**Keywords:** Nonlinear phenomena, Complex networks

## Abstract

Photochemically coupled Belousov-Zhabotinsky micro-oscillators are studied in experiments and simulations. Generally good agreement between the experimental and simulated dynamical behavior is found, with spiral wave chimeras exhibited at small values of the time delay in the coupling between the oscillators, spiral wave core splitting at higher values, and phase cluster states replacing the spiral wave dynamics at the highest values of the time delay. Spiral wave chimera dynamics is exhibited experimentally for much of the time delay range, while spiral wave phase cluster states are exhibited more in the model simulations. In addition to comparing the experimental and simulation behavior, we explore the novel spiral wave phase cluster states and develop a mechanism for this new and unusual dynamical behavior.

## Introduction

The spiral wave chimera in two-dimensional arrays of coupled oscillators is a remarkable spatiotemporal pattern, with an ordered spiral wave rotating around a core of incoherent oscillators. Discovered by Kuramoto and Shima^1–3^ in arrays of nonlocally coupled phase and FitzHugh-Nagumo oscillators, as well as in the complex Ginzburg-Landau equation, a number of theoretical studies of the spiral wave chimera state subsequently appeared^4–14^. Spiral wave chimeras have recently been studied experimentally by Totz *et al*.^15^ in large arrays of photochemically coupled Belousov-Zhabotinsky (BZ) oscillators.

In a theoretical study of spiral wave chimeras in arrays of phase oscillators, Martens *et al*.^4^ discuss several puzzles concerning the nature of these chimeras, noting that the state becomes numerically unobservable on increasing a phase-lag parameter in the model, and the bifurcation associated with this phenomenon is not known. Another puzzle is that the spiral wave chimera exists only for values of the phase-lag parameter that are a small fraction of the natural period. This is in contrast to chimera states in one-dimensional ring configurations of phase oscillators, which are found at values of the phase-lag parameter around $\pi \mathrm{/2}$^9,16,17^. Another unusual characteristic of spiral wave chimeras is their erratic motion arising from the asynchronous oscillators in the core.

While the dynamics of BZ oscillators is quite different from phase oscillator dynamics, there are striking similarities in the spiral wave chimeras in these systems. In the BZ spiral wave chimera^15^, a time delay is used in the coupling, which plays a role similar to the phase-lag parameter^18,19^. Like the phase oscillator system studied by Martens *et al*.^4^, the BZ spiral wave chimera also exists for time delays that are only a small fraction of the natural period. This is in contrast to one-dimensional BZ chimera states, which occur with time delays of approximately 3/4 of the period^6,20–22^.

In this paper, we present experimental and computational studies of the dependence of the dynamical behavior on the time delay used in the nonlocal coupling of the BZ oscillators. New insights into the underlying dynamics of the BZ spiral wave chimera^15^ are described. We computationally explore the range of coupling time delays for which the spiral wave chimera state exists and determine delay values where the character of the behavior changes. We characterize the dynamics of the remaining spiral wave, which now advances with pulsing behavior. On further increasing the time delay, the core is similar to the core of aperiodic oscillators of the spiral wave chimera in the sense that erratic motion is exhibited; however, phase clusters^23,24^ now occur in the core. In both experiments and simulations at higher values of time delay, spiral wave core splitting occurs. In both cases, this behavior is replaced by periodic phase clusters in the oscillator population. While there is good overall qualitative agreement between the experimental and simulation dynamical behavior, there are interesting differences in the transition to phase cluster behavior, with spiral wave chimera dynamics dominant in experiments and phase cluster dynamics dominant in simulations. Here, we focus primarily on the novel phase cluster dynamics found in our computational modeling study.

## Procedures

### Experimental

Our experimental studies of spiral wave chimeras utilize chemical oscillators prepared as in Totz *et al*.^15^ by loading cation-exchange beads with a photosensitive catalyst for the BZ reaction, ruthenium-tris-dimethylene-bipyridine (${{\rm{Ru}}({\rm{dmbpy}})}_{3}^{2+}$). The catalyst-loaded beads are positioned in a precision drilled acrylic plate, which is bathed in catalyst-free BZ reaction mixture. The behavior of the discrete oscillators is monitored in order to select the oscillators that form a reasonably narrow period distribution and to remove defective oscillators. These oscillators are indexed to form a virtual array of up to 40 × 40 = 1600 BZ oscillators.

The BZ oscillators are monitored by measuring the fluorescence, which is proportional to the concentration of ${{\rm{Ru}}({\rm{dmbpy}})}_{3}^{2+}$, allowing the state of each oscillator to be determined. The nonlocal coupling is carried out by illuminating oscillator (*j,k*), at the center of a square region of side length $2l+1$, according to its state and the states of its neighboring oscillators described by Eq. (1):

1 $${I}_{j,k}={I}_{0}+K\mathop{\sum }\limits_{m=j-l}^{j+l}\,\mathop{\sum }\limits_{n=k-l}^{k+l}{e}^{-\kappa r}[{g}_{m,n}(t-\tau )-{g}_{j,k}(t)].$$

The light intensity ${I}_{j,k}$ projected on oscillator (*j, k*) is equal to a background light intensity ${I}_{0}$ plus the difference between the state of oscillator (*j, k*), measured in gray value ${g}_{j,k}(t)$, and the states of its neighboring oscillators (*m, n*), measured in gray values ${g}_{m,n}(t-\tau )$, at times $t$ and ($t-\tau$), respectively. The coupling strength decays exponentially with the distance $r=\sqrt{{(m-j)}^{2}+{(n-k)}^{2}}$ between oscillators (*j, k*) and ($m,n$), which is governed by the decay constant $\kappa$. The actinic light at 440 nm is well separated from the measured fluorescence above 550 nm. The oscillatory period of oscillator (*j, k*) is determined by measuring the time interval between successive peaks in the time series of the oxidized catalyst concentration, which is proportional to the gray value ${g}_{j,k}$. The initial conditions are obtained by entraining each oscillator to a periodic external forcing with individual phase offsets ${\psi }_{j,k}$, which are given by ${\psi }_{j,k}=\text{arctan}((k-{k}_{0})/(j-{j}_{0}))$. The boundary conditions are specified in ref. ^15^ in Eqs. S1 and S2 (Supplementary Information), where the range of the nonlocal coupling function is modified to omit non-existent nodes beyond the grid.

### Computational

The kinetics of the BZ oscillators can be modeled with the dimensionless Zhabotinsky-Buchholtz-Kiyatkin-Epstein (ZBKE) model^25^, modified to describe the photosensitive ruthenium-catalyzed, coupled discrete oscillator system^20,26–28^:

2 $$\begin{array}{c}\frac{d{u}_{j,k}}{dt}=\frac{1}{{\varepsilon }_{1}}\left({I}_{j,k},+,\frac{\mu -{u}_{j,k}}{\mu +{u}_{j,k}},(\beta +\frac{\alpha {q}_{j,k}{v}_{j,k}}{{\varepsilon }_{3}+1-{v}_{j,k}}),+,\gamma ,{\varepsilon }_{2},{w}_{ss,j,k}^{2},+,(1,-,{v}_{j,k},),{w}_{ss,j,k},-,{u}_{j,k}^{2},-,{u}_{j,k}\right),\\ \,\frac{d{v}_{j,k}}{dt}=2{I}_{j,k}+(1-{v}_{j,k}){w}_{ss,j,k}-\frac{\alpha {v}_{j,k}}{{\varepsilon }_{3}+1-{v}_{j,k}},\\ {w}_{ss,j,k}=\frac{1}{4\gamma {\varepsilon }_{2}}(\sqrt{16\gamma {u}_{j,k}{\varepsilon }_{2}+{v}_{j,k}^{2}-2{v}_{j,k}+1}+{v}_{j,k}-1),\end{array}$$

where the variables ${u}_{j,k}(t)$ and ${v}_{j,k}(t)$ represent the concentrations of ${{\rm{HBrO}}}_{2}$ and ${{\rm{Ru}}({\rm{dmbpy}})}_{3}^{3+}$ on oscillator ($j,k$), and ${w}_{ss,j,k}({u}_{j,k},{v}_{j,k})$ represents the steady-state concentration of ${{\rm{HBrO}}}_{2}^{+}$. The stoichiometric parameter for oscillator ($j,k$) is ${q}_{j,k}=0.7\pm 0.2$, giving the average natural period and standard deviation ${T}_{0}=36.0\pm 1.6$, the time scale parameters are ${\varepsilon }_{1}=0.11$, ${\varepsilon }_{2}=1.7\times {10}^{-5}$, ${\varepsilon }_{3}=1.6\times {10}^{-3}$, the chemical kinetics parameters are $\alpha =0.1$, $\beta =1.7\times {10}^{-5}$, $\gamma =1.2$, $\mu =2.4\times {10}^{-4}$, and the simulation time step is $\Delta t=1.0\times {10}^{-4}$. The oscillatory period of oscillator ($j,k$) is determined by measuring the time interval between successive peaks in the time series of the oxidized catalyst concentration, ${v}_{{\rm{j}},{\rm{k}}}$. The initial conditions and boundary conditions are the same as in the experiments. In addition, simulations using periodic boundary conditions were also carried out, described in ref. ^15^ in Fig. S2 and Eq. S3 (Supplementary Information). There were no significant differences in the properties of the spiral wave chimera for the two types of boundary conditions.

Because the gray values in the coupling Eq. (1) are proportional to the oxidized catalyst concentration, we have an analogous coupling equation for the simulations in terms of ${[{\rm{Ru}}({\rm{dmbpy}})}_{3}^{3+}]$, which is represented by the variables ${v}_{j,k}(t)$ and ${v}_{m,n}(t-\tau )$ for oscillators ($j,k$) and ($m,n$) in Eq. (3):

3 $${I}_{j,k}={I}_{0}+K\mathop{\sum }\limits_{m=j-l}^{j+l}\,\mathop{\sum }\limits_{n=k-l}^{k+l}{e}^{-\kappa r}[{v}_{m,n}(t-\tau )-{v}_{j,k}(t)].$$

Here, ${I}_{j,k}$ is the simulation light intensity projected onto oscillator (*j, k*), ${I}_{0}=5.25\times {10}^{-4}$ is the background light intensity, $K=1.6\times {10}^{-4}$ is the coupling strength, $\kappa =0.4$ is the coupling decay constant, $l=4$ is the maximum coupling distance, and $\tau$ is the time delay.

We also define a two-dimensional local order parameter ${R}_{j,k}$, which permits delineation of the asynchronous core region from the synchronous spiral wave:

4 $${R}_{j,k}=\frac{1}{{(2\delta +1)}^{2}}|\mathop{\sum }\limits_{m=j-\delta }^{j+\delta }\,\mathop{\sum }\limits_{n=k-\delta }^{k+\delta }{e}^{i{\theta }_{m,n}}|,$$

where ${\theta }_{m,n}$ are the phases of oscillators (*m, n*) in a square region of side length $2\delta +1$, with oscillator (*j, k*) in the center.

## Computational Dynamical Behavior as a Function of Time Delay

**Figure 1:**
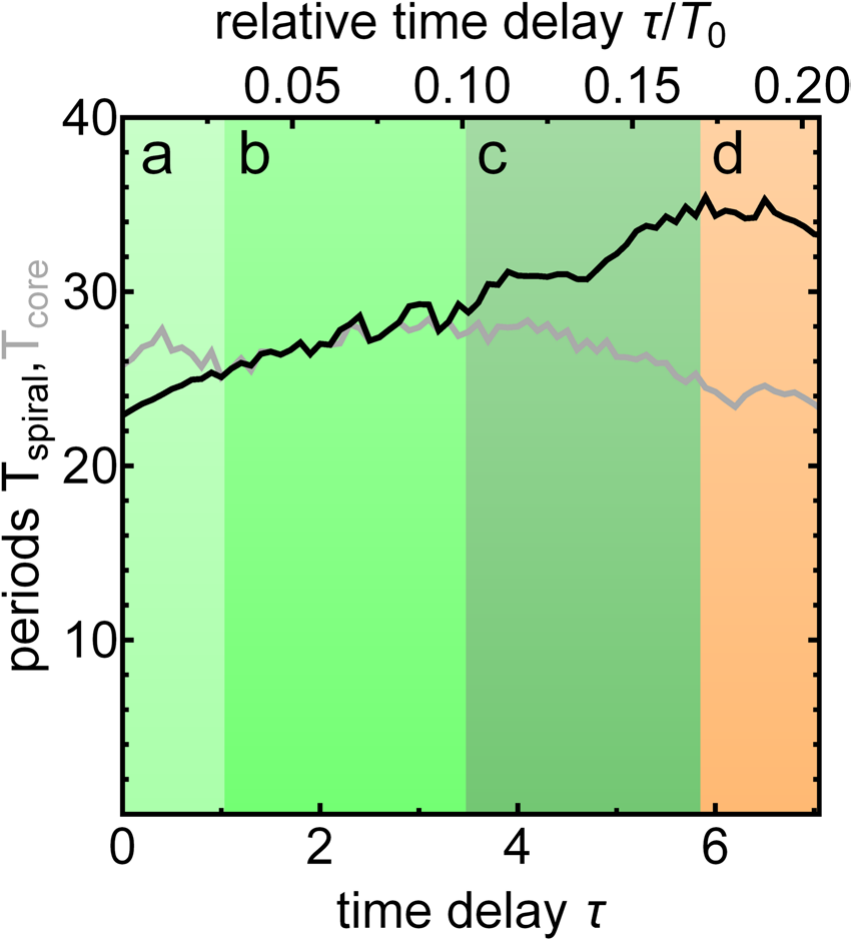
Period of oscillators making up the spiral wave (black line) and time-averaged period of oscillators in the core of the spiral wave (gray line) as a function of time delay $\tau$ and relative time delay $\tau /{T}_{0}$, where ${T}_{0}$ is the average natural period. The core oscillators are identified as the oscillators for which the local order parameter ${R}_{j,k} < 0.4$, as defined in Eq. (4). All simulations utilize the modified ZBKE model described by Eq. (2) in an array of $64\times 64$ oscillators with nonlocal coupling according to Eq. (3). Average natural period and standard deviation: ${T}_{0}=36.0\pm 1.6$.

As the value of the time delay $\tau$ is increased, the perturbation to oscillator ${v}_{j,k}(t)$ from oscillators ${v}_{m,n}(t-\tau )$ is increasingly delayed, as seen in Eq. (3). The effect of the delay is to lengthen the period of the spiral wave, becoming an approximately linear function of $\tau$, as shown in Fig. 1. Also shown in this figure is the time averaged period of the core oscillators as a function of $\tau$, which remains approximately constant for much of the range of $\tau$.

Four regions of dynamical behavior are exhibited in Fig. 1 : (a) At small values of time delay, $\tau \lesssim 1.0$ (light green), the spiral wave period is less than the averaged period of the spiral core; (b) at slightly larger values, $1.0\lesssim \tau \lesssim 3.5$ (green), the spiral wave period is approximately equal to the averaged spiral core period; (c) at still larger values, $3.5\lesssim \tau \lesssim 5.7$ (dark green), there is a range over which the spiral wave period is greater than the averaged period of the spiral core; (d) at the highest values of time delay, $\tau \mathop{ > }\limits_{\sim }5.7$ (orange), spiral wave core splitting occurs, which then is replaced by phase cluster behavior. The dynamical behavior in each of these regions is examined in the following sections.

### Spiral wave chimeras with $\tau \lesssim 1.0$

**Figure 2:**
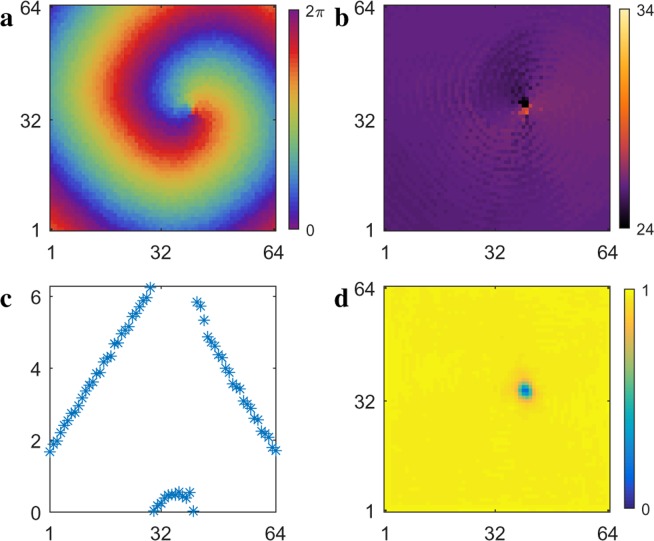
Spiral wave chimera in an array of $64\times 64$ oscillators with nonlocal coupling according to Eq. (3) and the time delay $\tau =0.75$. (**a**) Phase and (**b**) period of each oscillator, and (**d**) the local order parameter ${R}_{j,k}$ defined by Eq. (4). (**c**) Oscillators firing in horizontal cross section at oscillator 32, which can be compared with the advancing spiral wave in (**a**). See Supplementary Information for corresponding video. ${T}_{spiral}\approx 25$ and average ${T}_{core}\approx 26$.

At small values of the time delay, $\tau \lesssim 1.0$, the period of the spiral wave is smaller than the averaged period of the aperiodic oscillators in the spiral wave core (Fig. 1, light green). Figure 2 shows an example of a spiral wave chimera in this range, with $\tau =0.75$. The phase and period of each oscillator are shown in Fig. 2(a,b), and the local order parameter ${R}_{j,k}$ is shown in Fig. 2(d). The core of the spiral wave chimera is quite small; however, the larger averaged period of the core can be discerned as well as the lower value of the order parameter associated with the asynchronous oscillators. The phases $\theta$ of oscillators in a horizontal cross section at oscillator number 32 are shown in Fig. 2(c). We see the phases of the oscillators in the spiral wave regularly advance, although this regularity is disrupted when the core oscillators are sampled.

**Figure 3:**
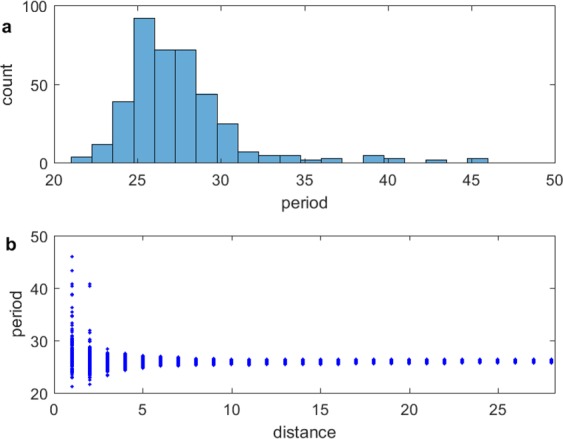
(**a**) Occurrence plot of number of oscillators with period $T$ for ${R}_{j,k} < 0.4$. (**b**) Period as a function of integer distance, which originates at the center of the spiral wave chimera core.

Shown in Fig. 3(a) is an occurrence plot of the number of oscillators with period $T$ over time in which the local order parameter ${R}_{j,k}\mathrm{ < \; 0.4}$. Figure 3(b) shows a plot of the period over time as a function of integer distance, which originates at the center of the core of the spiral wave chimera. A wide range of periods is exhibited for the oscillators within and near the core, while the range in period narrows in the spiral wave farther away from the core. The aperiodicity of the core oscillators can be discerned in Fig. 3(a,b).

### Collapse of the spiral wave chimera core for $1.0\lesssim \tau \lesssim 3.5$

**Figure 4:**
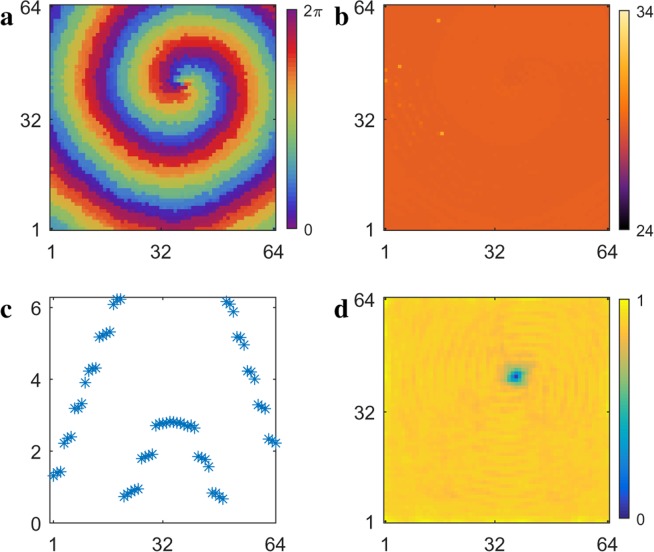
(**a**) Phase $\theta$, (**b**) period $T$, and (**d**) local order parameter ${R}_{j,k}$ of the oscillators for $\tau =3.3$. (**c**) Oscillators firing in horizontal cross section at oscillator 32, which can be compared with the advancing wave in (**a**). See Supplementary Information for corresponding video.

As shown in Fig. 1, the spiral wave period and the averaged period of the oscillators of the spiral wave core become approximately equal over a range of the time delay, $1.0\lesssim \tau \lesssim 3.5$ (green). The spiral wave core becomes smaller within this range of $\tau$ values until it virtually disappears, in the sense of the core comprising oscillators that differ from the spiral wave oscillators. Figure 4(a) shows the phase $\theta$ of each oscillator in the array for $\tau =3.3$. Figure 4(b,d) show the period $T$ and local order parameter ${R}_{j,k}$ of the oscillators. With the core essentially disappearing, the period is remarkably uniform and there is no evidence of oscillator aperiodicity in the system. The low value of the local order parameter shown in Fig. 4(d) arises from the phase singularity at the tip of the spiral wave in the oscillator array^29,30^.

Figure 4(c) shows the phases $\theta$ of oscillators in the horizontal cross section at oscillator number 32, where we see a new feature. The spiral wave originates at the tip, which is almost centered at oscillator number 32 on the x-axis. Close examination of the video of Fig. 4(a,c) reveals that the entire spiral wave advances in pulses, with each rotational period of the spiral made up of 7 pulses.

When the oscillators fire in the wave front, the time-delayed coupling gives rise to another collection of oscillators ahead of the wave front that fire at time $\tau {\prime}$ later. Here, the time $\tau {\prime}$ is defined as the time delay $\tau$ plus the time required for the oscillators to fire after being perturbed, $\tau {\prime} =\tau +1.1$, where the firing time of 1.1 is determined by the best overall fit. The firing of these oscillators gives rise, in turn, to yet another collection of oscillators ahead of the wave front firing, again at time $\tau {\prime}$ later. This process results in the spiral wave advancing one complete rotation in 7 pulses, with the period given by $T\,=7\times \tau {\rm{{\prime} }}\approx 31$. This pulsing structure defines the wave front of the spiral, which is first sampled as the counter-clockwise rotating spiral passes through the horizontal cross section on the left and again as it passes through the cross section on the right. Each time a group of oscillators fires in the wave front, they perturb oscillators ahead at time $\tau$ in the almost completely relaxed wave back. These oscillators are ready to fire and they do so at time $\tau {\prime} -\tau$ after the perturbation. Insights into the firing dynamics of BZ oscillators can be found by considering the phase response curves (PRC) of the system, which, for strongly perturbed oscillators, has a refractory region at early values of the perturbation phase and an almost instantaneous firing region at later values of the perturbation phase^15,22,31–35^. The pulsing structure of the spiral wave will be further characterized in the next section in which we define the various sequences of phase clusters found in the system.

### Phase cluster spiral wave cores for $3.5\lesssim \tau \lesssim 5.7$

**Figure 5:**
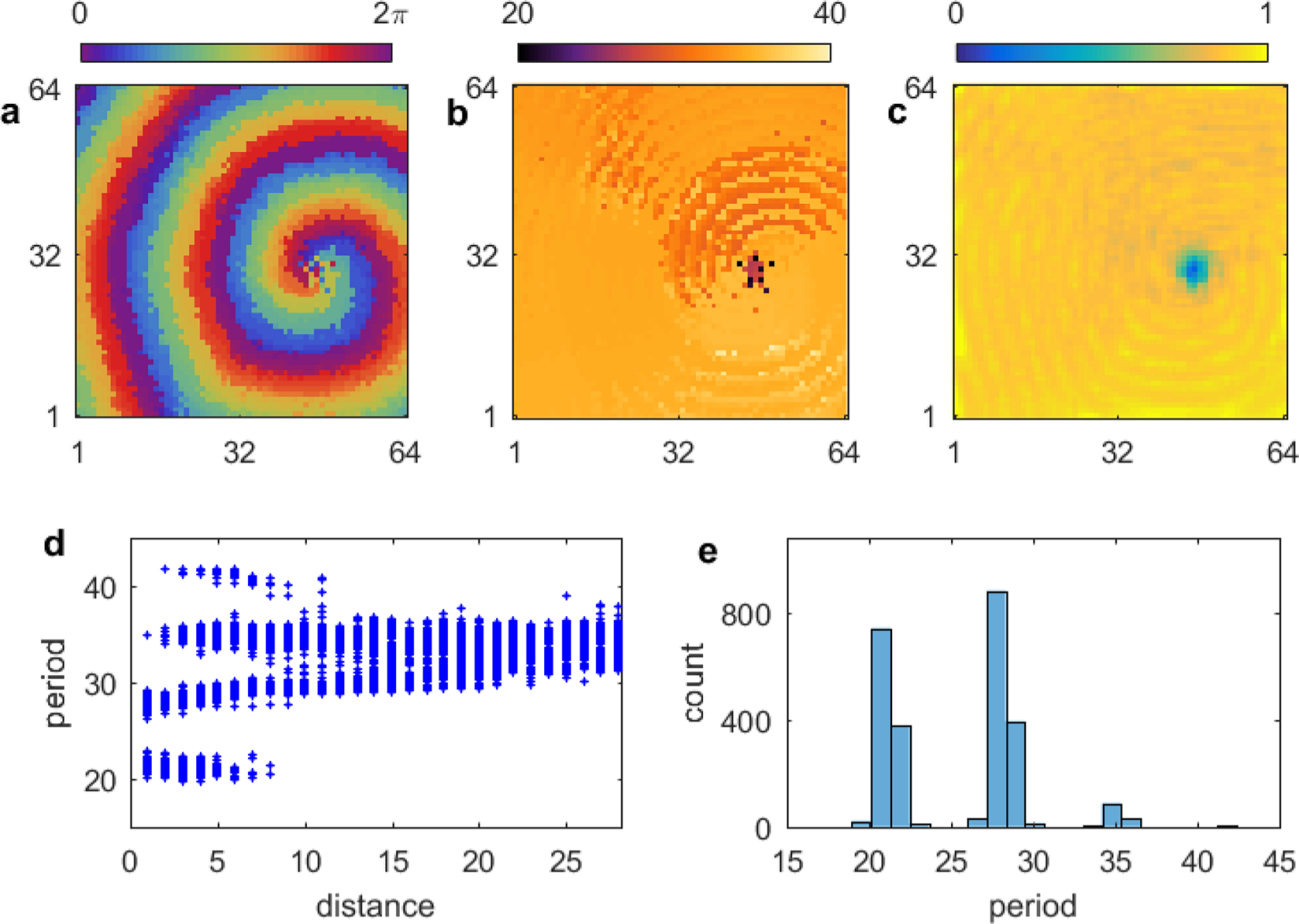
(**a**) Phase $\theta$, (b) period $T$, and (**c**) local order parameter ${R}_{j,k}$ of the oscillators for $\tau \,\mathrm{=\; 5.7}$, where the period of the spiral wave is larger than the time averaged period of the core of the spiral wave. (**d**) Period as a function of integer distance, which originates at the center of the spiral wave core. (**e**) Occurrence plot of the number of oscillators with period $T$ for ${R}_{j,k} < 0.4$. See Supplementary Information for corresponding video.

For values of the time delay $3.5\lesssim \tau \lesssim 5.7$, the spiral wave period is larger than the time averaged period of the oscillators in the spiral core. This change has significant consequences for the dynamical behavior of oscillators within the spiral core, which reappears and grows larger with increasing $\tau$. Figure 5(a) shows a plot of the phase of each oscillator in the array of coupled oscillators for $\tau \,\mathrm{=\; 5.7}$, which appears similar to the plots of oscillator phase for smaller values of $\tau$. However, the period $T$ and local order parameter ${R}_{j,k}$, shown respectively in Fig. 5(b,c), display features not previously observed. Much greater motion of the core now gives rise to large Doppler effects, where oscillators in the direction in which the core is moving fire earlier while those in the opposite direction fire later. This effect can also be seen in Fig. 5(d), in which the spiral wave exhibits a large period distribution even at distances far removed from the core. Another new feature is exhibited by the spiral core and in a radius of approximately 9 oscillators from the center of the core: phase cluster states firing with four different preferred periods. These groups of oscillators can be seen in Fig. 5(d) as well as in the occurrence plot in Fig. 5(e), which shows the number of oscillators with period $T$ over time for which the local order parameter ${R}_{j,k}\mathrm{ < 0.4}$. Each group of preferred period oscillators form a phase cluster state, with the number of clusters in the state determining the period.

Examination of the successive firing of oscillators in the spiral core and the surrounding region reveals that when a collection of oscillators fires, this gives rise to another, different collection of oscillators firing at time $\tau {\prime}$ later. The firing of these oscillators gives rise, in turn, to yet another, different collection of oscillators firing, again at time $\tau {\prime}$ later. This process continues until the firing of a collection of oscillators results in perturbing the original collection of oscillators to fire, giving rise to the beginning of a new period of oscillation. The time $\tau {\prime}$ is the same as earlier defined for the pulsing spiral wave shown in Fig. 4.

**Figure 6:**
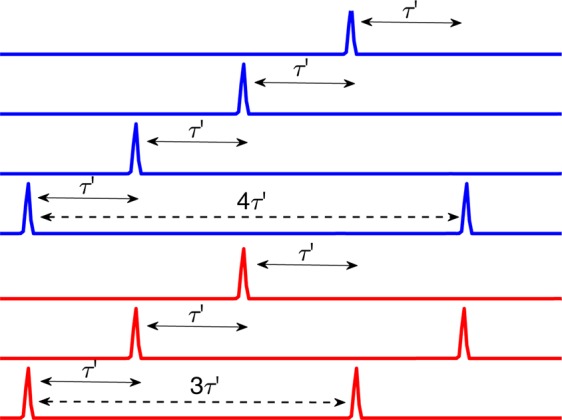
Schematic representation of successive firing of oscillators that gives rise to phase cluster states with distinct periods^22,33^. Shown are three-cluster (red) and four-cluster states (blue) that result in period $T=3\times \tau {\prime}$ and $T=4\times \tau {\prime}$ cluster states, corresponding to the first and second cluster states in Fig. 5(d,e). The time $\tau \text{'}$ is defined as $\tau \text{'}=\tau +1.1$, where $\tau$ is the time delay and 1.1 is the time required for an oscillator to fire after being perturbed.

Schematic representations of this process are shown in Fig. 6, where three-cluster and four-cluster states are shown in red and blue, respectively. The three-cluster state corresponds to the oscillators with period $T\approx 20$ shown in Fig. 5(d,e). The first collection of oscillators fires and $\tau {\prime}$ later the second collection of oscillators fires. This perturbation results in the third collection of oscillators firing at time $\tau {\prime}$ later, which results in the first collection of oscillators firing, beginning a new period of oscillation with $T\,\mathrm{=\; 3}\times \tau {\prime} \approx 20$. The four-cluster state, shown in blue, corresponds to the oscillators with period $T\approx 27$, shown in Fig. 5(d,e). Here, the time delay plus firing time $\tau \text{'}$ remains the same; however, now there is a fourth group of oscillators such that the period is $T\,\mathrm{=\; 4}\times \tau {\prime} \approx 27$.

As shown in Fig. 5(d), there are two more phase cluster states that produce periods given by $T\,=\,5\times \tau {\rm{{\prime} }}\approx 34$ and $T\,\mathrm{=\; 6}\times \tau {\prime} \approx 41$. Whereas the first two phase cluster states appear to be made up of oscillators randomly located in the spiral core and surroundings, the third phase cluster, which has the same period as the spiral wave, $T\approx 34$, corresponds to pulsing behavior, with 5 pulses per period. This pulsing behavior is much like the seven-cluster state shown in Fig. 4(d), with a spiral period of $T\mathrm{=\; 7}\times \tau {\prime} \approx 31$.

The fourth phase cluster state in Fig. 5(d) is unusual in the sense that its period $T\approx 41$ is larger than the average natural period of ${T}_{0}\,\mathrm{=\; 36.0}\pm 1.6$. The oscillators in this group are perturbed by the last group of oscillators firing in the $T\approx 34$ pulsing cluster state to yield a period $T\approx 41$ cluster state. The period is larger than the natural period ${T}_{0}$ apparently because the last group was previously perturbed at a phase that resulted in a phase delay and, hence, a larger period. We note that this group is very small in Fig. 5(d,e).

Even though there are four cluster states of oscillators, with four distinct periods, remarkably, these oscillators fire in unison each time increment of $\tau {\prime}$. The firing of oscillators in each cluster of a cluster state gives rise to oscillators firing in another cluster $\tau {\prime}$ later, either in the core or the relaxed wave back of the spiral wave ahead. The cross-talk between the clusters gives rise to synchronized firing of oscillators in the different cluster states, since oscillators firing in the wave front perturb not only the oscillators in their own cluster state but also oscillators in other cluster states. This results in the pulsing behavior of the entire spiral wave and core.

### Phase cluster behavior and spiral core splitting for $\tau \gtrsim 5.7$

**Figure 7:**
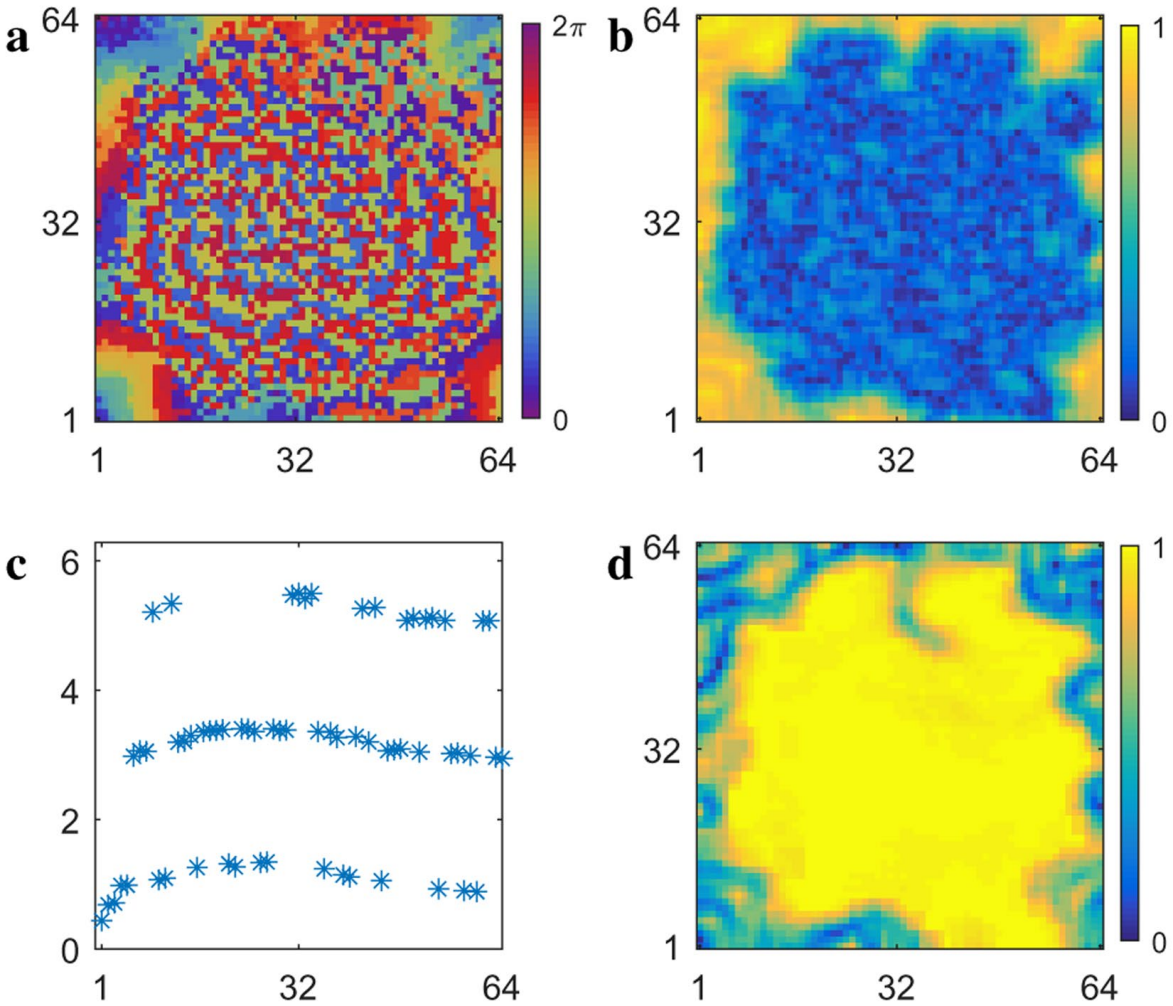
Phase cluster behavior. (**a**) Phase $\theta$ and (**b**) local order parameter ${R}_{j,k}$ of the oscillators for $\tau =6.6$. (**c**) Phase of oscillators firing in horizontal cross section at oscillator 32 as a function of oscillator index. (**d**) Local order parameter ${R}_{j,k}^{3}$ defined by Eq. (5) for the three-cluster state. See Supplementary Information for corresponding video.

The spiral waves with phase cluster cores are completely replaced by phase cluster behavior on increasing the value of the time delay to $\tau \gtrsim 6.5$. The phase cluster behavior for $\tau \,\mathrm{=\; 6.6}$ is shown in Fig. 7. The phases of the oscillators in the array are shown in Fig. 7(a), where propagating waves form phase clusters with the period $T=3\times \tau {\prime} \approx 23$. The local order parameter defined by Eq. (4) is very low, as shown in Fig. 7(b), since three different groups of oscillators successively firing give rise to one period of oscillation. Figure 7(c) shows a plot of oscillator phase at the oscillator 32 horizontal cross section as a function of oscillator index, in which three clusters of oscillators firing make up the phase cluster state.

5 $${R}_{j,k}^{d}=\frac{1}{{(2\delta +1)}^{2}}|\mathop{\sum }\limits_{m=j-\delta }^{j+\delta }\,\mathop{\sum }\limits_{n=k-\delta }^{k+\delta }{e}^{di{\theta }_{m,n}}|$$

The two-dimensional local order parameter ${R}_{j,k}$ in Eq. (4) is generalized for phase cluster states in Eq. (5), where $d$ is defined as an integer. The value of $d$ in the exponent reduces the period of the complex exponential. For example, for $d=2$, the phases ${\theta }_{j,k}=0$ and ${\theta }_{j,k}=\pi$ map to the same value, and therefore the value of ${R}^{2}$ is 1, corresponding to a two-cluster or antiphase state.

Figure 7(d) shows the local order parameter ${R}_{j,k}^{3}$ for three-cluster states^36^, defined by Eq. (5) for $d=3$. We see that the order given by ${R}_{j,k}^{3}$ is high, as the three groups of oscillators give rise to the phase cluster state. The period is defined by the successive firing of the three groups of oscillators or, equivalently, by the firing of each particular oscillator. We note that the phase cluster state described in Fig. 7 is unlike phase clusters in homogeneous oscillatory media, in which spatially distinct regions successively oscillate^37^, for example, three regions in a three-cluster state in the homogeneous spatiotemporal BZ system. The oscillators in the phase cluster state described in Fig. 7 are stochastically distributed through the oscillator array but are connected via the propagating wave.

**Figure 8:**
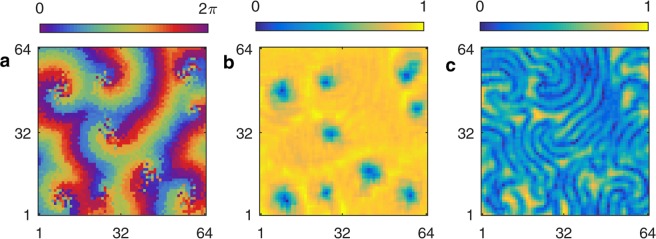
Spiral wave core splitting. (**a**) Phase of each oscillator in the oscillator array with multiple spiral waves for $\tau =6.2$. (**b**) Local order parameter ${R}_{j,k}$ defined by Eq. (4) for one-group phase clusters and (**c**) local order parameter ${R}_{j,k}^{3}$ defined by Eq. (5) for three-group phase clusters. See Supplementary Information for corresponding video.

Finally, it is instructive to return to the spiral waves with phase-cluster cores to examine the phenomenon of core splitting^15^. Figure 8 shows the system at *τ*  = 0.62, where multiple splitting events have given rise to a continually fluctuating state of multiple spiral waves having phase cluster cores. This state is similar to the vortex glass consisting of regular spiral waves observed by Brito *et al*.^38^. The oscillator phase in the array of oscillators is shown in Fig. 8(a), and the order parameters ${R}_{j,k}$ and ${R}_{j,k}^{3}$ are shown in Fig. 8(b,c). Again, we see low order in the spiral wave cores and high order in the surrounding spiral waves according to ${R}_{j,k}$ in Fig. 8(b). We see that the larger spiral wave cores exhibit high values in the order parameter ${R}_{j,k}^{3}$ in Fig. 8(c), corresponding to the high order of the three-cluster state.

We note that the splitting of the spiral core is qualitatively different from previously observed spiral instabilities^39,40^. Core splitting behavior occurs as the core grows large enough that the spiral wave tip cannot circumnavigate the core before a wave segment spontaneously appears ahead of the tip. The spiral tip connects to the segment and continues around the large core; however, the core continues to grow and the spontaneously generated wave segment grows further into the core each spiral wave period. By the time the spiral wave tip is making its third or forth trip around the core, the core splits into two cores, typically one with two spiral tips and a topological charge of Q = +2 and the other with one spiral tip and a topological charge of Q = −1. The core fragment with two spiral tips quickly splits into two spirals with cores, each with topological charge of Q = +1, while the other spiral wave maintains its topological charge of Q = −1. Hence, the topological charge of Q = +1 is maintained throughout the splitting process, from the original spiral wave with topological charge of Q = +1. Formally, the topological charge can be determined with Eq. (6), where $\varDelta {\phi }_{k}$ are the phase differences between oscillators on a closed curve $\gamma$^41^.

6 $${\rm{Q}}=\frac{1}{2\pi }{\oint }_{\gamma }\phi ds=\sum _{k}\,{\rm{mod}}(\Delta {\phi }_{k}\mathrm{,2}\pi )$$

## Comparing Computational and Experimental Dynamical Behavior

The intricate phase cluster behavior exhibited in our simulations prompted us to further examine the experimental dynamical behavior as a function of time delay $\tau$. Experimental phase cluster behavior similar to the simulation phase clusters shown in Fig. 7 was found in our original investigation^15^ at values of $\tau$ larger than those corresponding to the spiral wave core splitting.

**Figure 9:**
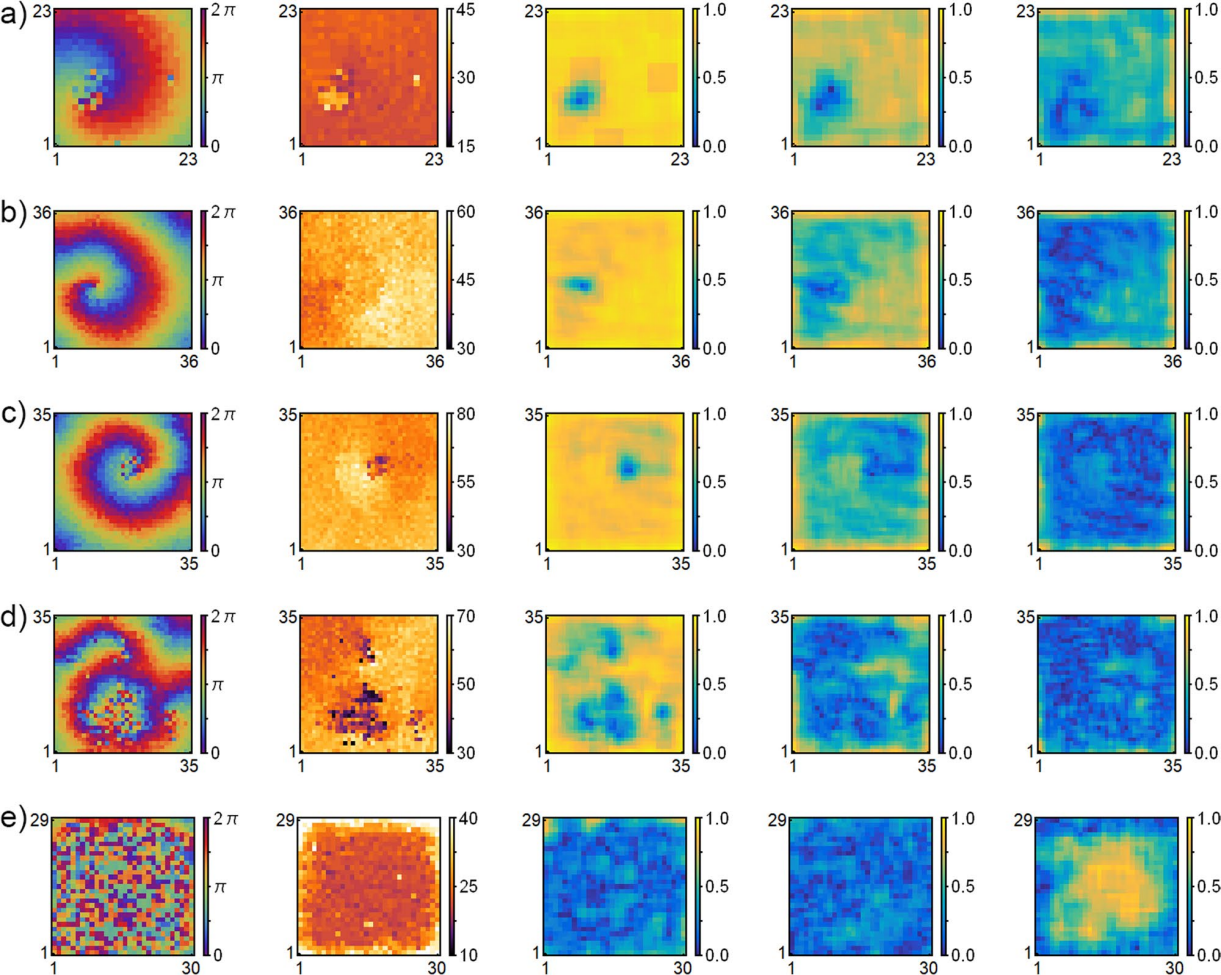
Phase cluster analysis of experimental spiral wave chimeras (**a**–**c**), spiral wave core splitting (**d**), and phase clusters (**e**). Five different experiments showing column wise, respectively, phase, period and the order according to 3 different order parameters, ${R}_{j,k}^{1}$, ${R}_{j,k}^{2}$, and ${R}_{j,k}^{3}$, defined as in Eqs. (4) and (5). Experimental parameters: (**a**) time delay $\tau =1.0$ s, mean period and standard deviation ${T}_{0}=54.8\pm 1.4$ s, $\tau /{T}_{0}=0.018$, coupling constant $K=0.15$, coupling decay constant $\kappa =0.3$; (**b**) $\tau =3.0$ s, ${T}_{0}=114.8\pm 8.2$ s, $\tau /{T}_{0}=0.026,\,K=0.10$, $\kappa =0.4$; (**c**) $\tau =5.0$ s, ${T}_{0}=142.2\pm 11.1$ s, $\tau /{T}_{0}=0.035,\,K=0.125$, $\kappa =0.40$; (**d**) $\tau =5.0$ s, ${T}_{0}=84.8\pm 8.1$ s, $\tau /{T}_{0}=0.059,\,K=0.08$, $\kappa =3.1$; (**e**) $\tau =5.0$ s, ${T}_{0}=54.7\pm 7.1$ s, $\tau /{T}_{0}=0.091,\,K=0.08$, $\kappa =0.32$.

We are able to examine the virtual array in our experiments for evidence of phase cluster behavior in a manner similar to the order parameter analysis in simulations, shown in Figs. 7 and 8. An order parameter analysis of experiments with spiral wave chimeras, spiral core splitting and phase cluster dynamics is shown in Fig. 9. Each experiment has five panels, which respectively show phase, period and three local order parameters, ${R}_{j,k}^{1}$ – ${R}_{j,k}^{3}$, defined as in Eqs. (4) and (5). The local order parameters were used to search for one-cluster, two-cluster and three-cluster states.

As can be seen in Fig. 9(a–c), the value of each order parameter remains small in the spiral wave chimera core, with no evidence of phase clusters. As expected, the value of ${R}_{j,k}^{1}$ is high for the spiral wave in each of the experiments, reflecting the higher order. These results indicate that spiral wave chimeras with aperiodic core oscillators occur in the experiments over a wider range of time delay $\tau$ than in the simulations. Because the natural period varies from experiment to experiment, we use the relative time delay $\tau /{T}_{0}$ in comparisons of experimental and simulated dynamical behavior. (See Fig. 1 for relative time delay $\tau /{T}_{0}$ in the simulations.)

We find that spiral wave chimeras are not observed above $\tau /{T}_{0}=\mathrm{1.0/36.0}=0.026$ in simulations, while experimental chimera dynamics is observed for values of $\tau /{T}_{0}=\mathrm{5.0/142.2}=0.035$. Spiral wave core splitting is experimentally observed at $\tau /{T}_{0}=\mathrm{5.0/84.8}=0.059$, which compares with $\tau /{T}_{0}=\mathrm{6.2/36.0}=0.172$ for this behavior in simulations. Spiral wave behavior is completely replaced by phase clusters in both the experiments and simulations at higher values of $\tau /{T}_{0}$, with three-cluster states found at $\tau /{T}_{0}=\mathrm{5.0/54.7}=\mathrm{0.0.91}$ in experiments, Fig. 9(e), and the same cluster behavior found at $\tau /{T}_{0}=\mathrm{6.6/36.0}=0.183$ in simulations, Fig. 7(d). Hence, the appearance of phase cluster dynamics occurs in about half the range of $\tau /{T}_{0}$ in experiments compared to the range of $\tau /{T}_{0}$ in simulations.

**Figure 10:**
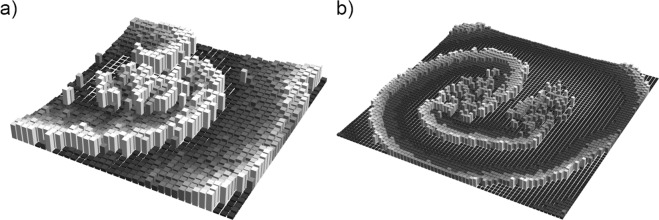
Spiral wave pulsing dynamics, where the third dimension represents the concentration of the oxidized BZ catalyst, ${{\rm{Ru}}({\rm{dmbpy}})}_{3}^{3+}$. (**a**) Experimental example of spiral wave exhibiting pulsing dynamics. Parameters: (**a**) $\tau =5.0$ s, ${T}_{0}=142.2\pm 11.1$ s, $\tau /{T}_{0}=0.035,K=0.125$, $\kappa =0.40$. (**b**) Simulation example of spiral wave exhibiting pulsing dynamics. Parameters: ${T}_{0}=36.0$, $\tau =5.9$, $\tau /{T}_{0}=\mathrm{5.9/36.0}=0.163$, $K=5.25\times {10}^{-4}$, $\kappa =0.40$. See Supplementary Information for corresponding videos.

We also searched for experimental spiral wave pulsing behavior such as that found in the simulations, as shown in Fig. 4. Figure 10 shows 3D representations of spiral wave core splitting in an experiment and a simulation, where the third dimension represents the concentration of the oxidized BZ catalyst, ${{\rm{Ru}}({\rm{dmbpy}})}_{3}^{3+}$. In the experimental example, shown in Fig. 10(a), mild periodic pulsing of the spiral wave can be discerned in the video. In the simulation example, shown in Fig. 10(b), very strong pulsing can be observed in the video.

Although the spiral wave chimera behavior is significantly more robust in the experiments than in the simulations, phase cluster dynamics is exhibited by both at higher values of $\tau /{T}_{0}$. Figure 8 shows that phase cluster behavior also plays a role in spiral core splitting in simulations, and the pulsing behavior corresponds to the period of the spiral wave, given by $T=5\times \tau {\prime} \approx 37$, much like the pulsing spiral behavior in Fig. 5. While the pulsing behavior in the experimental spiral wave core splitting in Fig. 10(a) is much milder, it is indicative of pulsing spiral wave behavior similar to that seen in the simulations. In addition, a three-cluster state occurs at higher values of $\tau /{T}_{0}$, as shown in Fig. 9(d).

## Discussion

In both experiments and simulations, spiral wave chimera behavior is replaced by phase cluster dynamics following spiral wave core splitting. Spiral wave chimeras are dominant in the experiments, occurring in about 60% of the $\tau /{T}_{0}$ range before spiral wave core splitting. In simulations, phase cluster dynamics dominates, with chimeras appearing for only about 16% of the $\tau /{T}_{0}$ range before spiral wave core splitting. A potentially important observation is that when classic chimera behavior is observed in simulations the period of the spiral wave is always less than the averaged period of the spiral wave chimera core, as shown in Fig. 1. When these periods become equal, the spiral wave chimera core essentially disappears and periodic pulsing behavior is observed. It may be that the spiral wave chimera exists in simulations only when the spiral wave tip perturbs the core oscillators more frequently than their averaged period, such that aperiodic core oscillators are maintained. This is not the case in experiments, as Fig. 9(a–c) show the time averaged period of the spiral wave chimera core greater than, approximately equal to, and less than the spiral wave period, respectively.

We do not know the origin of the phase cluster dominance in our computational modeling results. A speculative possibility is that the dynamics of the two-variable ZBKE model, Eq. (2), is affected by large differences in time scales, making the model overly stiff to quantitatively agree with the experimental behavior. However, there is good qualitative agreement between experiment and simulation on the transition of spiral wave chimera dynamics to phase cluster dynamics, with spiral wave core splitting observed during the transition in both. In addition, the phase cluster spiral wave behavior found in our simulations represents novel spatiotemporal dynamics that, to our knowledge, has not been previously reported. We note that the simulation cluster behavior shown at $\tau =5.7$ in Fig. 5(d), where four phase cluster states with distinct periods near and inside the spiral core coexist with the spiral wave, is a type of chimera. It is not the classic chimera with a spiral wave having a core comprising aperiodic oscillators; however, it represents two coexisting spatiotemporal dynamical states that have significantly different properties. We believe that the good overall agreement between experiments and simulations, as well as the differences in the detailed dynamics, will be useful in developing a better understanding of the transition from spiral wave chimeras to phase cluster dynamics.

## Supplementary information

Supplementary Information.

Supplementary Information2.

Supplementary Information3.

Supplementary Information4.

Supplementary Information5.

Supplementary Information6.

Supplementary Information7.

Supplementary Information8.

## Data Availability

All data generated or analyzed during this study are included in this published article and its Supplementary Information files. The numerical simulation code for the computational results is available on a public Git repository: https://github.com/bzjan/Spiral_Wave_Chimera_Solver.git.
