# Supplementary material for: Transition from spiral wave chimeras to phase cluster states
Source: Sci Rep. 2020 May 8;10:7821. doi: 10.1038/s41598-020-64081-6 (PMC7210287; doi:10.1038/s41598-020-64081-6)
Supplement: Supplementary file 1 — Supplementary Information. [file 41598_2020_64081_MOESM1_ESM.pdf]

# Supplementary Information

# Transition from spiral wave chimeras to phase cluster states

**Jan Frederik Totz[1,2,4], Mark R. Tinsley[3], Harald Engel[4], and Kenneth Showalter[3]**

[1]Department of Mechanical Engineering, Massachusetts Institute of Technology, Cambridge, MA 02139, USA
[2]Department of Mathematics, Massachusetts Institute of Technology, Cambridge, MA 02142, USA
[3]C. Eugene Bennett Department of Chemistry, West Virginia University, Morgantown, WV 26506-6045 USA
[4]Institut für Theoretische Physik EW 7-1, TU Berlin, Hardenbergstr. 36, 10623 Berlin, Germany

# Supplementary Videos

### Fig. 2 Supplementary Video S2

Spiral wave chimera in an array of $64 \times 64 = 4096$ oscillators with nonlocal coupling according to Eq. (3) and the time delay $\tau = 0.75$. (a) Phase and (b) period of each oscillator, and (d) the local order parameter $R_{j,k}$ defined by Eq. (4). (c) Oscillators firing in horizontal cross section at oscillator 32, which can be compared with the advancing spiral wave in (a). $T_{spiral} \approx 25$ and average $T_{core} \approx 26$.

### Fig. 4 Supplementary Video S4

(a) Phase $\theta$, (b) period $T$, and (d) local order parameter $R_{j,k}$ of the oscillators for $\tau = 3.3$. (c) Oscillators firing in horizontal cross section at oscillator 32, which can be compared with the advancing wave in (a).

### Fig. 5 Supplementary Video S5

(a) Phase $\theta$, (b) period $T$, and (c) local order parameter $R_{j,k}$ of the oscillators for $\tau = 5.7$, where the period of the spiral wave is larger than the time averaged period of the core of the spiral wave. (d) Period as a function of integer distance, which originates at the center of the spiral wave core. (e) Occurrence plot of the number of oscillators with period $T$ for $R_{j,k} < 0.4$.

### Fig. 7 Supplementary Video S7

Phase cluster behavior. (a) Phase $\theta$ and (b) local order parameter $R_{j,k}$ of the oscillators for $\tau = 6.6$. (c) Phase of oscillators firing in horizontal cross section at oscillator 32 as a function of oscillator index. (d) Local order parameter $R^3_{j,k}$ defined by Eq. (5) for three phase cluster states.

### Fig. 8 Supplementary Video S8

Spiral wave core splitting. (a) Phase of each oscillator in the oscillator array with multiple spiral waves for $\tau = 6.2$. (b) Local order parameter $R_{j,k}$ defined by Eq. (4) for one phase cluster states and (c) local order parameter $R^3_{j,k}$ defined by Eq. (5) for three phase cluster states.

### Fig. 10 Supplementary Videos S10(a) and S10(b)

Spiral wave pulsing dynamics, where the third dimension represents the concentration of the oxidized BZ catalyst, $\mathrm{Ru(dmbpy)}_3^{3+}$. (a) Experimental example of spiral wave exhibiting pulsing dynamics. Parameters: $\tau = 5.0$ s, $T_0 = 142.2 \pm 11.1$ s, $\tau/T_0 = 0.035, K = 0.125$, $\kappa = 0.40$. (b) Simulation example of spiral wave exhibiting pulsing dynamics. Parameters: $\tau = 5.9$, $T_0 = 36.0$, $\tau/T_0 = 5.9/36.0 = 0.163$, $K = 5.25 \times 10^{-4}$, $\kappa = 0.40$.
